# Supplementary material for: Diet, Physical Activity and Adiposity as Determinants of Circulating Amino Acid Levels in a Multiethnic Asian Population
Source: Nutrients. 2020 Aug 27;12(9):2603. doi: 10.3390/nu12092603 (PMC7551953; doi:10.3390/nu12092603)
Supplement: Supplementary file 1 [file nutrients-12-02603-s001.pdf]

Table S1 Associations[1] between intake of red meat and poultry[2], and serum amino acid levels[3]. Significant associations and interactions following adjustment for multiple testing (adjusted *P* < 0.05) are in bold.

	Red meat (energy %)			Poultry (energy %)		
	Overall	Males	Females	Overall	Males	Females
Alanine	-0.058	-0.046	-0.054	-0.038	-0.053	-0.023
95% CI	(-0.265, 0.150)	(-0.357, 0.265)	(-0.335, 0.227)	(-0.263, 0.187)	(-0.407, 0.301)	(-0.316, 0.271)
P-interaction		0.732			0.971	
Arginine	-0.019	-0.067	0.042	0.085	0.136	0.057
95% CI	(-0.226, 0.203)	(-0.390, 0.256)	(-0.248, 0.331)	(-0.147, 0.318)	(-0.232, 0.504)	(-0.246, 0.35)
P-interaction		0.608			0.663	
Citrulline	-0.065	-0.140	0.004	-0.071	-0.087	-0.058
95% CI	(-0.265, 0.136)	(-0.445, 0.164)	(-0.264, 0.272)	(-0.288, 0.147)	(-0.433, 0.260)	(-0.338, 0.222)
P-interaction		0.266			0.826	
Glutamate/Glutamine	-0.053	-0.026	0.134	-0.029	-0.067	-0.005
95% CI	(-0.235, 0.129)	(-0.335, 0.284)	(-0.124, 0.393)	(-0.245, 0.187)	(-0.419, 0.285)	(-0.275, 0.265)
P-interaction		0.279			0.905	
Glycine	-0.089	-0.158	0.011	-0.177	-0.119	-0.188
95% CI	(-0.300, 0.123)	(-0.418, 0.103)	(-0.312, 0.334)	(-0.406, 0.053)	(-0.416, 0.178)	(-0.525, 0.150)
P-interaction		0.976			0.262	
Histidine	0.007	0.033	0.009	-0.076	0.029	-0.156
95% CI	(-0.208, 0.222)	(-0.290, 0.355)	(-0.281, 0.298)	(-0.309, 0.158)	(-0.338, 0.396)	(-0.458, 0.146)
P-interaction		0.943			0.195	
Isoleucine/Leucine	-0.053	-0.113	0.014	0.107	0.040	0.162
95% CI	(-0.235, 0.129)	(-0.400, 0.174)	(-0.217, 0.244)	(-0.090, 0.304)	(-0.287, 0.366)	(-0.079, 0.402)
P-interaction		0.291			0.955	
Methionine	0.029	0.039	0.028	0.011	0.130	-0.066
95% CI	(-0.176, 0.233)	(-0.281, 0.359)	(-0.236, 0.292)	(-0.211, 0.232)	(-0.235, 0.494)	(-0.342, 0.209)
P-interaction		0.816			0.225	
Ornithine	-0.022	-0.093	0.077	-0.093	-0.211	0.004
95% CI	(-0.225, 0.181)	(-0.400, 0.214)	(-0.194, 0.347)	(-0.313, 0.127)	(-0.560, 0.138)	(-0.278, 0.286)
P-interaction		0.203			0.455	
Phenylalanine	0.114	0.018	0.203	0.016	0.031	-0.023
95% CI	(-0.091, 0.319)	(-0.301, 0.336)	(-0.065, 0.470)	(-0.206, 0.239)	(-0.331, 0.393)	(-0.303, 0.257)
P-interaction		0.317			0.596	
Proline	-0.090	-0.169	-0.013	-0.171	-0.330	-0.045
95% CI	(-0.295, 0.114)	(-0.475, 0.137)	(-0.290, 0.264)	(-0.393, 0.051)	(-0.678, 0.018)	(-0.334, 0.244)
P-interaction		0.402			0.211	
Serine	0.028	-0.009	0.055	0.069	0.111	0.058
95% CI	(-0.185, 0.242)	(-0.306, 0.288)	(-0.251, 0.362)	(-0.163, 0.301)	(-0.227, 0.449)	(-0.262, 0.378)
P-interaction		0.665			0.981	
Tyrosine	0.048	-0.054	0.120	0.122	0.164	0.087
95% CI	(-0.158, 0.254)	(-0.367, 0.259)	(-0.154, 0.394)	(-0.101, 0.345)	(-0.193, 0.520)	(-0.199, 0.372)
P-interaction		0.235			0.579	
Valine	-0.084	-0.293	0.116	0.063	-0.128	0.213
95% CI	(-0.276, 0.109)	(-0.584, -0.001)	(-0.140, 0.371)	(-0.146, 0.272)	(-0.460, 0.205)	(-0.053, 0.480)
P-interaction		**0.027**			0.252	
Aromatic	0.064	-0.003	0.127	0.025	0.095	-0.039
95% CI	(-0.145, 0.273)	(-0.318, 0.313)	(-0.151, 0.406)	(-0.201, 0.252)	(-0.265, 0.454)	(-0.330, 0.252)
P-interaction		0.411			0.313	
Branched-chain	-0.074	-0.229	0.078	0.083	-0.063	0.199
95% CI	(-0.260, 0.113)	(-0.516, 0.058)	(-0.166, 0.321)	(-0.119, 0.285)	(-0.390, 0.264)	(-0.055, 0.454)
P-interaction		0.065			0.449	

[1] Adjusted for age, sex, ethnicity, body mass index, smoking status, alcohol drinking status, moderate-to-vigorous physical activity, and education level

[2] β-coefficients correspond to the change in amino acid levels associated with a 100% increase, or doubling, in daily intake of a given dietary source

[3] Amino acid concentrations were converted into *Z*-scores and are expressed per-SD

Table S2 Associations[1] between intake of seafood and soy[2], and serum amino acid levels[3]. Significant associations and interactions following adjustment for multiple testing (adjusted $P < 0.05$) are in bold.

	Seafood (energy %)			Soy (energy %)		
	Overall	Males	Females	Overall	Males	Females
Alanine	-0.061	-0.191	0.036	0.211	0.256	0.214
95% CI	(-0.201, 0.078)	(-0.410, 0.028)	(-0.146, 0.218)	(-0.366, 0.787)	(-0.676, 0.119)	(-0.522, 0.950)
P-interaction		0.082			0.950	
Arginine	0.007	0.018	-0.001	0.221	-0.032	0.412
95% CI	(-0.137, 0.152)	(-0.209, 0.246)	(-0.189, 0.186)	(-0.374, 0.816)	(-0.999, 0.935)	(-0.345, 1.170)
P-interaction		0.814			0.442	
Citrulline	**-0.168**	-0.164	-0.168	0.300	0.334	0.313
95% CI	**(-0.302, -0.033)**	(-0.379, 0.051)	(-0.341, 0.006)	(-0.257, 0.856)	(-0.578, 1.246)	(-0.388, 1.015)
P-interaction		0.951			0.915	
Glutamate/Glutamine	-0.018	-0.119	0.065	-0.278	-0.364	-0.142
95% CI	(-0.152, 0.116)	(-0.337, 0.099)	(-0.102, 0.232)	(-0.830, 0.274)	(-1.290, 0.563)	(-0.820, 0.535)
P-interaction		0.078			0.573	
Glycine	-0.051	-0.093	0.001	0.479	0.049	0.801
95% CI	(-0.194, 0.091)	(-0.277, 0.091)	(-0.208, 0.210)	(-0.109, 1.067)	(-0.731, 0.830)	(-0.044, 1.645)
P-interaction		0.263			0.197	
Histidine	-0.077	-0.062	-0.084	0.270	0.091	0.492
95% CI	(-0.222, 0.067)	(-0.289, 0.166)	(-0.271, 0.103)	(-0.327, 0.867)	(-0.875, 1.056)	(-0.266, 1.250)
P-interaction		0.665			0.392	
Isoleucine/Leucine	0.095	-0.011	**0.179**	-0.265	-0.521	0.014
95% CI	(-0.027, 0.217)	(-0.213, 0.192)	**(0.030, 0.328)**	(-0.769, 0.240)	(-1.381, 0.338)	(-0.590, 0.618)
P-interaction		**0.026**			0.160	
Methionine	0.084	0.078	0.101	-0.326	-0.476	-0.162
95% CI	(-0.053, 0.222)	(-0.148, 0.303)	(-0.070, 0.272)	(-0.892, 0.241)	(-1.434, 0.482)	(-0.854, 0.530)
P-interaction		0.590			0.457	
Ornithine	0.093	0.068	0.130	0.444	0.424	0.485
95% CI	(-0.044, 0.229)	(-0.148, 0.284)	(-0.045, 0.305)	(-0.119, 1.006)	(-0.494, 1.342)	(-0.223, 1.192)
P-interaction		0.239			0.615	
Phenylalanine	0.040	0.119	-0.014	-0.134	-0.346	0.106
95% CI	(-0.099, 0.178)	(-0.105, 0.343)	(-0.188, 0.159)	(-0.703, 0.435)	(-1.298, 0.607)	(-0.596, 0.808)
P-interaction		0.626			0.385	
Proline	-0.016	-0.081	0.029	**0.676**	0.724	0.647
95% CI	(-0.154, 0.122)	(-0.297, 0.135)	(-0.150, 0.208)	**(0.109, 1.244)**	(-0.192, 1.640)	(-0.077, 1.371)
P-interaction		0.308			0.932	
Serine	0.044	0.039	0.043	0.361	-0.109	0.690
95% CI	(-0.010, 0.188)	(-0.171, 0.248)	(-0.155, 0.242)	(-0.232, 0.954)	(-0.998, 0.780)	(-0.113, 1.492)
P-interaction		0.838			0.244	
Tyrosine	0.016	-0.063	0.072	-0.313	-0.434	-0.125
95% CI	(-0.123, 0.155)	(-0.283, 0.158)	(-0.105, 0.249)	(-0.884, 0.258)	(-1.370, 0.503)	(-0.842, 0.592)
P-interaction		0.269			0.458	
Valine	**0.149**	0.099	**0.194**	-0.351	-0.667	-0.073
95% CI	**(0.019, 0.278)**	(-0.107, 0.305)	**(0.029, 0.359)**	(-0.884, 0.183)	(-1.541, 0.207)	(-0.743, 0.596)
P-interaction		0.164			0.157	
Aromatic	-0.013	-0.012	-0.011	-0.064	-0.270	0.199
95% CI	(-0.154, 0.127)	(-0.235, 0.210)	(-0.191, 0.170)	(-0.644, 0.515)	(-1.215, 0.675)	(-0.530, 0.929)
P-interaction		0.617			0.314	
Branched-chain	**0.132**	0.057	**0.194**	-0.327	-0.630	-0.040
95% CI	**(0.006, 0.257)**	(-0.145, 0.260)	**(0.037, 0.352)**	(-0.845, 0.190)	(-1.489, 0.229)	(-0.678, 0.598)
P-interaction		0.074			0.140	

[1] Adjusted for age, sex, ethnicity, body mass index, smoking status, alcohol drinking status, moderate-to-vigorous physical activity, and education level

[2] β-coefficients correspond to the change in amino acid levels associated with a 100% increase, or doubling, in daily intake of a given dietary source

[3] Amino acid concentrations were converted into *Z*-scores and are expressed per-SD

Table S3 Associations between dietary protein intake[1] and serum amino acid levels[2] adjusted for demographic variables. Statistically significant associations following adjustment for multiple testing (adjusted $P < 0.05$) are in bold.

	Total protein (energy %)	Total meat and seafood (energy %)	Red meat (energy %)	Poultry (energy %)	Seafood (energy %)	Soy (energy %)
Alanine<br>*P*-value	-0.018 (-0.117, 0.081)<br>0.718	-0.025 (-0.124, 0.073)<br>0.613	-0.017 (-0.225, 0.190)<br>0.869	0.017 (-0.209, 0.243)<br>0.885	-0.051 (-0.192, 0.091)<br>0.482	0.253 (-0.327, 0.833)<br>0.393
Arginine<br>*P*-value	0.007 (-0.094, 0.109)<br>0.886	0.003 (-0.099, 0.104)<br>0.961	-0.039 (-0.252, 0.173)<br>0.716	0.054 (-0.178, 0.285)<br>0.650	0.003 (-0.142, 0.148)<br>0.972	0.168 (-0.427, 0.762)<br>0.581
Citrulline<br>*P*-value	**-0.119 (-0.214, -0.024)**<br>**0.014**	**-0.126 (-0.221, -0.031)**<br>**0.009**	-0.087 (-0.286, 0.112)<br>0.393	-0.132 (-0.350, 0.085)<br>0.234	**-0.167 (-0.303, -0.031)**<br>**0.016**	0.268 (-0.291, 0.826)<br>0.347
Glutamate/Glutamine<br>*P*-value	0.356 (-0.609, 1.321)<br>0.469	0.421 (-0.542, 1.383)<br>0.392	1.374 (-0.650, 3.397)<br>0.183	0.657 (-1.551, 2.865)<br>0.560	-0.031 (-1.413, 1.350)<br>0.965	-0.229 (-0.796, 0.338)<br>0.429
Glycine<br>*P*-value	-0.100 (-0.201, 0.002)<br>0.054	**-0.113 (-0.214, -0.012)**<br>**0.029**	-0.153 (-0.366, 0.059)<br>0.157	**-0.257 (-0.489, -0.025)**<br>**0.030**	-0.060 (-0.205, 0.085)<br>0.418	0.468 (-0.127, 1.064)<br>0.123
Histidine<br>*P*-value	-0.047 (-0.148, 0.055)<br>0.367	-0.055 (-0.156, 0.046)<br>0.285	0.002 (-0.211, 0.214)<br>0.989	-0.091 (-0.322, 0.141)<br>0.443	-0.078 (-0.223, 0.066)<br>0.288	0.299 (-0.295, 0.894)<br>0.324
Isoleucine/Leucine<br>*P*-value	**0.091 (0.003, 0.180)**<br>**0.043**	**0.097 (0.009, 0.185)**<br>**0.031**	0.029 (-0.157, 0.214)<br>0.760	0.192 (-0.011, 0.394)<br>0.063	0.111 (-0.015, 0.238)<br>0.084	-0.216 (-0.736, 0.303)<br>0.415
Methionine<br>*P*-value	0.051 (-0.045, 0.147)<br>0.297	0.059 (-0.037, 0.155)<br>0.228	0.046 (-0.156, 0.247)<br>0.656	0.036 (-0.184, 0.256)<br>0.748	0.086 (-0.052, 0.223)<br>0.221	-0.281 (-0.846, 0.283)<br>0.329
Ornithine<br>*P*-value	0.056 (-0.040, 0.152)<br>0.252	0.043 (-0.053, 0.138)<br>0.379	0.027 (-0.174, 0.228)<br>0.793	-0.073 (-0.292, 0.146)<br>0.513	0.104 (-0.033, 0.241)<br>0.136	0.447 (-0.116, 1.009)<br>0.120
Phenylalanine<br>*P*-value	0.074 (-0.024, 0.172)<br>0.141	0.076 (-0.022, 0.174)<br>0.127	0.166 (-0.039, 0.372)<br>0.113	0.078 (-0.147, 0.302)<br>0.496	0.049 (-0.091, 0.189)<br>0.494	-0.099 (-0.676, 0.477)<br>0.736
Proline<br>*P*-value	-0.031 (-0.127, 0.066)<br>0.538	-0.049 (-0.146, 0.047)<br>0.318	-0.075 (-0.278, 0.128)<br>0.469	-0.152 (-0.374, 0.069)<br>0.178	-0.007 (-0.145, 0.132)<br>0.924	**0.656 (0.087, 1.225)**<br>**0.024**
Serine<br>*P*-value	0.033 (-0.068, 0.134)<br>0.524	0.022 (-0.079, 0.123)<br>0.665	-0.005 (-0.217, 0.207)<br>0.966	0.030 (-0.201, 0.261)<br>0.800	0.036 (-0.108, 0.181)<br>0.622	0.359 (-0.234, 0.953)<br>0.236
Tyrosine<br>*P*-value	0.070 (-0.030, 0.169)<br>0.170	0.077 (-0.022, 0.176)<br>0.129	0.106 (-0.102, 0.315)<br>0.318	0.207 (-0.021, 0.434)<br>0.075	0.028 (-0.114, 0.170)<br>0.701	-0.262 (-0.847, 0.322)<br>0.379
Valine<br>*P*-value	**0.104 (0.010, 0.198)**<br>**0.030**	**0.111 (0.018, 0.205)**<br>**0.020**	0.001 (-0.196, 0.198)<br>0.992	0.167 (-0.048, 0.382)<br>0.127	**0.163 (0.029, 0.297)**<br>**0.017**	-0.273 (-0.824, 0.278)<br>0.332
Aromatic<br>*P*-value	0.036 (-0.063, 0.135)<br>0.480	0.036 (-0.063, 0.135)<br>0.474	0.105 (-0.102, 0.313)<br>0.320	0.077 (-0.149, 0.304)<br>0.504	-0.005 (-0.147, 0.137)<br>0.944	-0.017 (-0.600, 0.565)<br>0.954
Branched-chain<br>*P*-value	**0.102 (0.011, 0.193)**<br>**0.028**	**0.109 (0.019, 0.2.00)**<br>**0.018**	0.013 (-0.178, 0.204)<br>0.897	0.183 (-0.025, 0.391)<br>0.085	**0.148 (0.017, 0.278)**<br>**0.026**	-0.259 (-0.794, 0.276)<br>0.343

[1] β-coefficients correspond to the change in amino acid levels associated with a 100% increase, or doubling, in daily intake of a given dietary source
[2] Amino acid concentrations were converted to *Z*-scores and are expressed per-SD

Table S4 Associations between quartiles of moderate-to-vigorous physical activity[1] and serum amino acid levels[2] adjusted for demographic variables, using the lowest quartile as the reference category. Significant trends following adjustment for multiple testing (adjusted $P < 0.05$) are in bold.

	Quartile 1[3]	Quartile 2[4]	Quartile 3[5]	Quartile 4[6]	*P*-value for trend[7]
Alanine<br>95% CI	0.000<br>(reference)	-0.080<br>(-0.176, 0.016)	-0.075<br>(-0.171, 0.021)	-0.210<br>(-0.308, -0.113)	**0.002**
Arginine<br>95% CI	0.000<br>(reference)	-0.001<br>(-0.010, 0.098)	-0.160<br>(-0.259, -0.061)	-0.057<br>(-0.156, 0.043)	0.398
Citrulline<br>95% CI	0.000<br>(reference)	-0.012<br>(-0.105, 0.081)	-0.009<br>(-0.102, 0.084)	0.027<br>(-0.067, 0.121)	0.137
Glutamate/Glutamine<br>95% CI	0.000<br>(reference)	-0.082<br>(-0.176, 0.013)	-0.099<br>(-0.193, -0.005)	-0.118<br>(-0.214, -0.023)	0.704
Glycine<br>95% CI	0.000<br>(reference)	0.077<br>(-0.022, 0.176)	-0.015<br>(-0.114, 0.084)	0.014<br>(-0.086, 0.114)	0.482
Histidine<br>95% CI	0.000<br>(reference)	0.060<br>(-0.039, 0.158)	0.036<br>(-0.063, 0.135)	-0.047<br>(-0.147, 0.052)	0.098
Isoleucine/Leucine<br>95% CI	0.000<br>(reference)	-0.044<br>(-0.130, 0.043)	-0.018<br>(-0.104, 0.068)	-0.123<br>(-0.210, -0.036)	**0.037**
Methionine<br>95% CI	0.000<br>(reference)	-0.014<br>(-0.108, 0.080)	-0.055<br>(-0.149, 0.039)	-0.105<br>(-0.200, -0.010)	0.082
Ornithine<br>95% CI	0.000<br>(reference)	-0.081<br>(-0.174, 0.013)	-0.089<br>(-0.179, 0.008)	0.005<br>(-0.089, 0.099)	0.078
Phenylalanine<br>95% CI	0.000<br>(reference)	-0.034<br>(-0.130, 0.062)	-0.015<br>(-0.111, 0.081)	-0.084<br>(-0.181, 0.013)	0.491
Proline<br>95% CI	0.000<br>(reference)	-0.083<br>(-0.177, 0.011)	-0.124<br>(-0.218, -0.029)	-0.190<br>(-0.285, -0.095)	**0.002**
Serine<br>95% CI	0.000<br>(reference)	-0.018<br>(-0.117, 0.080)	-0.103<br>(-0.202, -0.005)	-0.129<br>(-0.229, -0.030)	0.070
Tyrosine<br>95% CI	0.000<br>(reference)	-0.055<br>(-0.152, 0.042)	-0.076<br>(-0.173, 0.021)	-0.081<br>(-0.179, 0.017)	0.456
Valine<br>95% CI	0.000<br>(reference)	-0.010<br>(-0.102, 0.081)	0.001<br>(-0.091, 0.092)	-0.089<br>(-0.182, 0.003)	0.062
Aromatic<br>95% CI	0.000<br>(reference)	-0.010<br>(-0.106, 0.087)	-0.022<br>(-0.119, 0.075)	-0.086<br>(-0.183, 0.012)	0.191
Branched-chain<br>95% CI	0.000<br>(reference)	-0.024<br>(-0.113, 0.064)	-0.007<br>(-0.096, 0.082)	-0.106<br>(-0.196, -0.016)	**0.043**

[1] β-coefficients correspond to the change in amino acid levels associated with moving from the first MVPA quartile to successive quartiles
[2] Amino acid concentrations were converted to *Z*-scores and are expressed per-SD
[3] Quartile 1: < 8.75 MET-hrs/wk
[4] Quartile 2: 8.75 – 22.74 MET-hrs/wk
[5] Quartile 3: 22.75 – 54.99 MET-hrs/wk
[6] Quartile 4: ≥ 55.00 MET-hrs/wk
[7] *P*-value calculated using the Wilcoxon signed-rank test

Table S5 Associations between anthropometric measures[1] and serum amino acid levels[2] adjusted for demographic variables. Significant associations following adjustment for multiple testing (adjusted $P < 0.05$) are in bold.

	BMI (kg/m^2)	Waist circumference (cm)
Alanine	**0.035**	**0.015**
95% CI	**(0.026, 0.044)**	**(0.012, 0.019)**
P-value	**< 0.001**	**< 0.001**
Arginine	**-0.014**	-0.004
95% CI	**(-0.023, -0.005)**	(-0.007, 0.000)
P-value	**0.002**	0.073
Citrulline	**-0.017**	**-0.006**
95% CI	**(-0.026, -0.009)**	**(-0.010, -0.003)**
P-value	**< 0.001**	**< 0.001**
Glutamate/Glutamine	**0.062**	**0.024**
95% CI	**(0.054, 0.071)**	**(0.021, 0.028)**
P-value	**< 0.001**	**< 0.001**
Glycine	**-0.045**	**-0.016**
95% CI	**(-0.054, -0.036)**	**(-0.020, -0.013)**
P-value	**< 0.001**	**< 0.001**
Histidine	**-0.012**	-0.002
95% CI	**(-0.021, -0.003)**	(-0.005, 0.002)
P-value	**0.007**	0.289
Isoleucine/Leucine	**0.055**	**0.021**
95% CI	**(0.048, 0.063)**	**(0.018, 0.024)**
P-value	**< 0.001**	**< 0.001**
Methionine	0.007	0.004
95% CI	(-0.001, 0.016)	(0.001, 0.007)
P-value	0.088	0.052
Ornithine	**0.019**	**0.007**
95% CI	**(0.011, 0.027)**	**(0.003, 0.010)**
P-value	**< 0.001**	**< 0.001**
Phenylalanine	**0.045**	**0.016**
95% CI	**(0.037, 0.054)**	**(0.012, 0.019)**
P-value	**< 0.001**	**< 0.001**
Proline	**0.024**	**0.011**
95% CI	**(0.016, 0.033)**	**(0.007, 0.014)**
P-value	**< 0.001**	**< 0.001**
Serine	**-0.029**	**-0.011**
95% CI	**(-0.038, -0.020)**	**(-0.014, -0.007)**
P-value	**< 0.001**	**< 0.001**
Tyrosine	**0.060**	**0.022**
95% CI	**(0.052, 0.069)**	**(0.019, 0.026)**
P-value	**< 0.001**	**< 0.001**
Valine	**0.061**	**0.023**
95% CI	**(0.053, 0.069)**	**(0.020, 0.026)**
P-value	**< 0.001**	**< 0.001**
Aromatic	**0.037**	**0.014**
95% CI	**(0.028, 0.045)**	**(0.011, 0.018)**
P-value	**< 0.001**	**< 0.001**
Branched-chain	**0.061**	**0.023**
95% CI	**(0.053, 0.069)**	**(0.020, 0.026)**
P-value	**< 0.001**	**< 0.001**

[1] β-coefficients correspond to the change in amino acid levels associated with a one-unit increment in body mass index and waist circumference

[2] Amino acid concentrations were converted to *Z*-scores and are expressed per-SD
